# Ambulance Transport Intervals for Children and Adults With Anaphylaxis: A Retrospective Analysis

**DOI:** 10.7759/cureus.87819

**Published:** 2025-07-13

**Authors:** Aina Takeuchi, Shinya Takeuchi, Marina Minami, Taku Oishi, Kingo Nishiyama, Mikiya Fujieda

**Affiliations:** 1 Pediatrics, Kochi Medical School, Kochi University, Nankoku, JPN; 2 Disaster and Emergency Medicine, Kochi Medical School, Kochi University, Nankoku, JPN; 3 Integrated Center for Advanced Medical Technologies, Kochi Medical School, Kochi University, Nankoku, JPN

**Keywords:** ambulances, anaphylaxis, children, japan, transportation of patients

## Abstract

Background: Anaphylaxis is a potentially fatal condition that can cause respiratory or cardiac arrest within 30 minutes. Therefore, it is important to shorten the interval from the emergency call to hospital arrival as much as possible. However, in rural areas, ambulance transport intervals may be greater for children than for adults with anaphylaxis because of the lack of nearby pediatric medical facilities. Thus, we aimed to compare ambulance transport intervals between children and adults with anaphylaxis.

Methods: This retrospective observational study used data from the Kochi-Iryo-Net database. We included patients with anaphylaxis who were transported to the emergency department between April 1, 2015, and March 31, 2021. Children were defined as those aged <15 years. The primary outcome measure was the total time required for ambulance transportation, consisting of dispatch time (from call to on-site arrival), on-site time (from arrival to departure), and travel time (from departure to hospital arrival). To adjust for patient background, we performed multiple linear regression analyses including age group, sex, illness severity, and ambulance department location.

Results: During the study period, 797 patients with anaphylaxis were transported to the emergency department, among whom 155 (19.4%) were children. There was no significant difference in the total ambulance transport interval (children: 31 minutes vs. adults: 32 minutes, p = 0.41). However, the interval from on-site departure to hospital arrival was five minutes longer for children (16 minutes vs. adults: 11 minutes, p < 0.01).

Conclusion: While no significant difference was observed in the total transport interval between children and adults, pediatric patients experienced longer travel times. Enhancing pediatric transport logistics, such as expanding access to pediatric-capable facilities or revising Emergency Medical Services triage and destination protocols, may help reduce this delay.

## Introduction

Anaphylaxis is a potentially fatal condition that can result in respiratory or cardiac arrest [1-3]. The lifetime prevalence of anaphylaxis is estimated to be 0.3%-5.1% [4,5]. In children, the incidence of anaphylaxis is reported to be 1-761 per 100,000 person-years [6]. In the early stages of anaphylaxis, it is difficult to predict the speed of progression and eventual severity, and death may occur within minutes [7]. Early administration of intramuscular adrenaline, including through autoinjectors, is the most critical intervention. While some guidelines suggest that patients with rapid response to epinephrine may not require hospital transport, this is not widely practiced in Japan, where most cases are still transported to emergency departments [8]. Therefore, it is important to shorten the interval from the emergency call to hospital arrival as much as possible.

The treatment for anaphylaxis is early intramuscular injection of adrenaline. In Japan, the rate of adrenaline autoinjectors (AAIs) or adrenaline administered by bystanders increased from 7% in 2013 to 27% in 2018 [9], although most patients did not receive adrenaline before hospital admission. Furthermore, acceptance of children with anaphylaxis at general hospitals may be delayed, as adrenaline dosage varies by body weight and not all facilities are equipped to manage pediatric emergencies. In many cases, barriers exist not only in dosage calculation but also in the overall capacity of emergency departments to provide pediatric-specific assessment, monitoring, and airway management. Therefore, emergency transportation acceptance of these cases may take longer than for adults. Particularly in rural areas, access to a central location may take longer, further delaying responses.

To the best of our knowledge, no studies have compared transport times between children and adults with anaphylaxis to the emergency department. Furthermore, no studies have compared the temporal segments within the transport process for cases of anaphylaxis, such as the interval from emergency call to on-site arrival, duration of on-site stay, and the interval between on-site departure and hospital arrival. Therefore, the objective of this study was to compare ambulance transport times, including total transport time and its component intervals, between children and adults with anaphylaxis.

This article was previously posted to the Research Square preprint server on December 24, 2024.

## Materials and methods

Ethics approval

This study was approved by the Ethical Review Committee of Kochi University School of Medicine (ERB-107870). Informed consent was obtained in the form of an opt-out clause on the Kochi-Iryo-Net’s website.

Study design

This retrospective observational study used data from the Kochi-Iryo-Net, Kochi Prefecture’s emergency medical and wide-area disaster information systems. The Kochi Prefecture is a rural area in Japan with a total population of 691,527 (2020 census) and an area of 7,104 km^2^ [10]. In the center, 47.2% of the population (326,545 people) live in Kochi City, with 10.8% aged <15 years (74,946 children) [10]. When the ambulance team transports patients to the emergency department, they enter the information into the Kochi-Iryo-Net database. The ambulance teams use dedicated tablets to accurately record various time-related data, including the total transport interval, interval from the emergency call to on-site arrival, on-site interval, and interval from on-site departure to hospital arrival. The medical institution’s destination, severity, and up to three diagnoses are recorded by the physician who treats the patient. The Kochi Prefecture compiled all data. We extracted data on emergency transport from the Kochi-Iryo-Net database.

The study period was from April 1, 2015, to March 31, 2021. Patients diagnosed with anaphylaxis were included in the study. We defined children as those aged <15 years, by the standard pediatric classification used in Japanese healthcare and statistical systems. The ages were categorized into five-year increments as per the data provided by the Kochi Prefecture. Patients with missing data were excluded.

Data collection and measurements

The primary outcome measure was the total time required for ambulance transportation, defined as the interval from the emergency call to hospital arrival. The secondary outcomes were the interval from the emergency call to on-site arrival, the on-site interval, and the interval from on-site departure to hospital arrival. We investigated patient characteristics such as sex, illness severity, and location of ambulance departments. Illness severity was classified as either hospitalization or nonhospitalization. The locations of the ambulance departments were categorized as Kochi City (the most urban area) or others.

Statistical analyses

The median and interquartile range were calculated for each interval, and the number of cases and percentages were calculated for the nominal variables. As the data were nonnormally distributed, Mann-Whitney U tests were used to compare each interval. Fisher’s exact test was used to compare group differences in sex distribution, ambulance department, and severity of illness.

Multiple linear regression analysis for the primary outcome and outcomes with statistically and clinically significant differences was performed to identify factors influencing patient characteristics. Clinically significant differences in terms of time intervals were defined as at least five minutes based on a previous study [7]. The independent variables were children (adults serving as the reference), location of the ambulance department (others serving as the reference), sex (females serving as the reference), and illness severity (non-hospitalization serving as the reference). Independent variables were selected based on a priori hypotheses.

Statistical significance was set at a two-tailed p value of <0.05. All data analyses were performed using EZR software, version 1.33 (Saitama Medical Center, Jichi Medical University, Saitama, Japan), which is a graphical user interface for R (The R Foundation for Statistical Computing, Vienna, Austria). Specifically, it is a modified version of the R Commander designed to add statistical functions frequently used in biostatistics [11].

## Results

**Figure 1:**
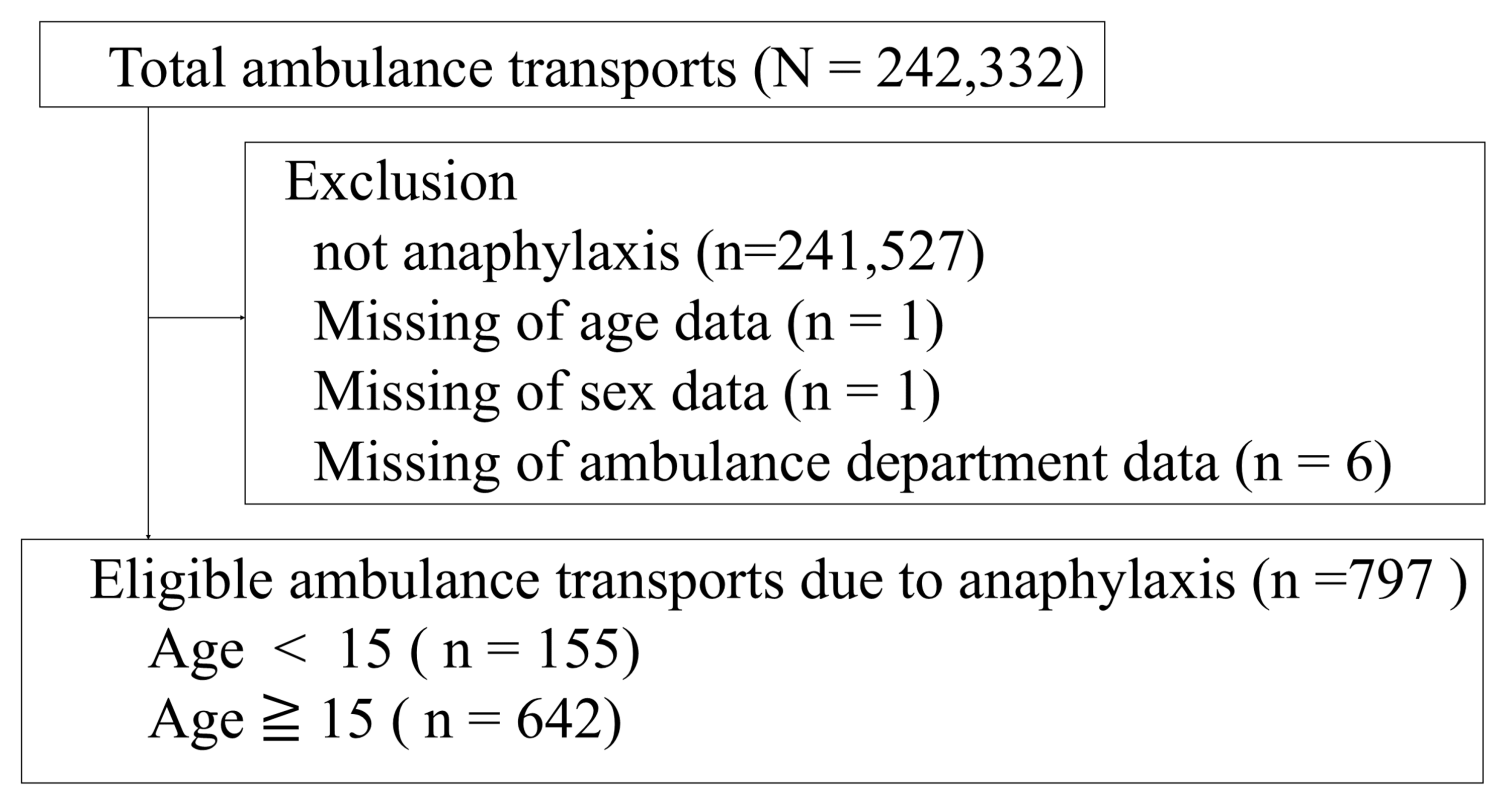
Selection of ambulance transportation for patients with anaphylaxis Patients were included between April 1, 2015, and March 31, 2021

During the six-year study period, 242,332 ambulance transportations and 805 anaphylactic cases were recorded. We excluded eight cases with missing data; thus, the data of 797 cases were extracted for analysis (Figure 1). Of these, 155 (19.4%) were children. There were no deaths due to anaphylaxis during the study period.

The characteristics of ambulance transport are summarized in Table 1.

**Table 1:** Characteristics of ambulance transport for anaphylaxis during the study period ^*^Data on sex, disposition, and department are presented as n (%). p values were calculated using Fisher’s exact test for categorical variables with small expected counts. No test statistic is reported for Fisher’s exact test as it does not yield a numerical value like χ² ^**^Transport time data are presented as median (IQR). The Mann-Whitney U tests were used for nonparametric continuous variables ^†^Test statistic values (χ² or U) are reported in the second-to-last column ^††^A two-tailed p value of <0.05 was considered statistically significant N/A: not available; IQR: interquartile range

Parameters	Pediatric patients (age <15 years)	Adult patients (age ≥15 years)	Test statistic^†^	p value^††^
Total	155	642	-	-
Sex^*^, n (%)				
Male	97 (62.6)	378 (58.9)	N/A	0.41
Disposition^*^, n (%)				
Hospitalization	94 (60.6)	387 (60.3)	N/A	1.00
Department^*^, n (%)				
Kochi City	73 (47.1)	239 (37.2)	N/A	0.03
Median transport time^**^, median (IQR)				
Total transport time	31 (25-39.5)	32 (25-43)	W = 51,863	0.41
Time from emergency call to arrival at the site	7 (5-9.5)	8 (6-11)	W = 58,066	<0.01
Time to stay at the site	7 (5-10)	10 (7-14)	W = 67,303	<0.01
Time from the site departure to the hospital arrival	16 (11-22)	11 (6-19)	W = 37,965	<0.01

**Figure 2:**
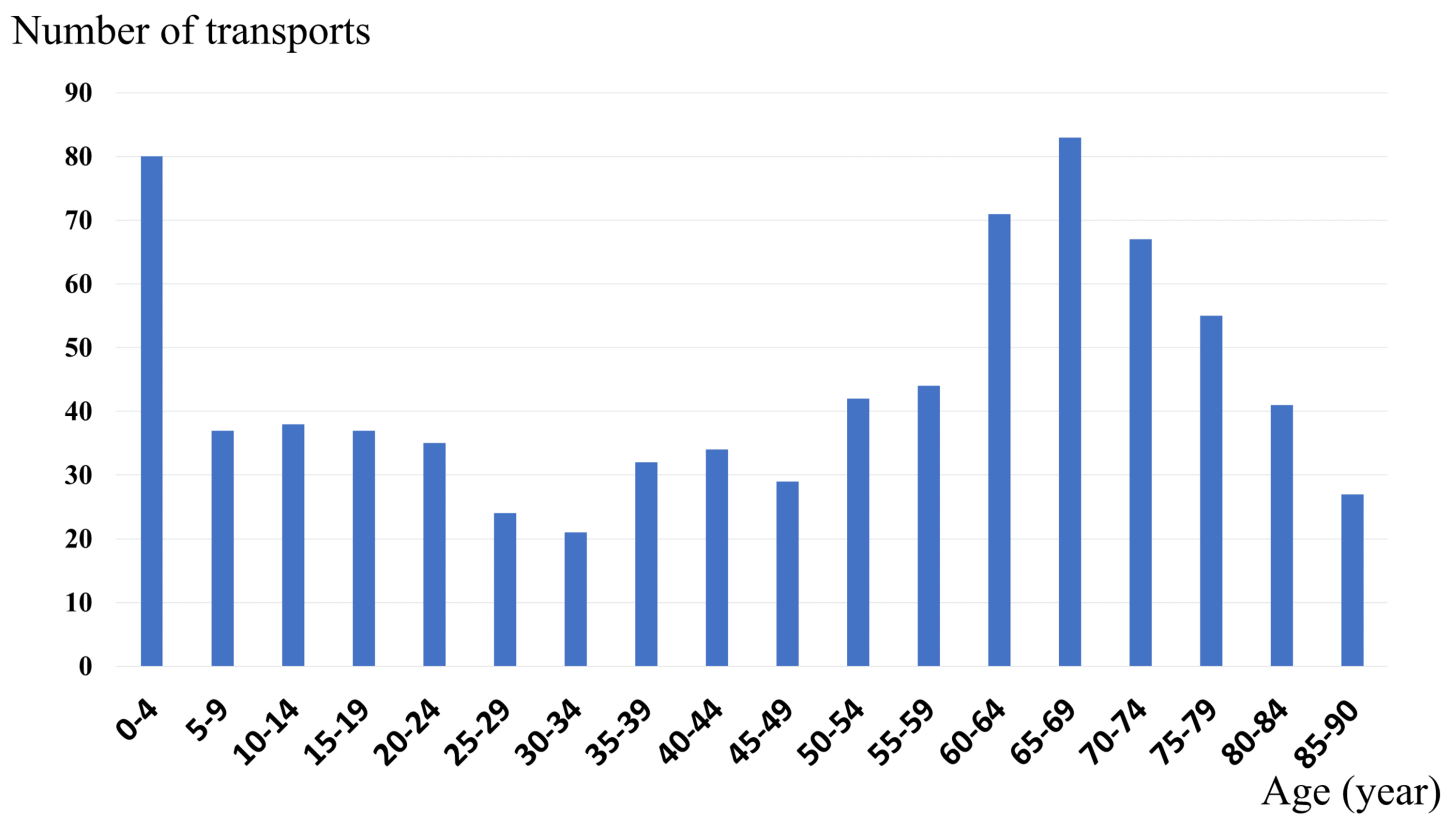
Number of patients with anaphylaxis transported by ambulance by age category The ages are categorized in five-year increments, as per the data provided by the Kochi Prefecture

There were no significant differences in sex or illness severity between children and adults. However, children were more frequently transported from Kochi City than adults (73 children, 47.1% vs. 239 adults, 37.2%; p = 0.03). The most common age group was 65-69 years, followed by the 0-4 years age group (Figure 2).

In unadjusted comparisons, there was no significant difference in total transport time between children and adults (31 vs. 32 minutes; p = 0.41). However, children had shorter on-site intervals (7 vs. 10 minutes; p < 0.01) and significantly longer travel times from on-site departure to hospital arrival (16 vs. 11 minutes; p < 0.01) (Table 1).

Multiple linear regression analysis showed that pediatric patients were independently associated with a 4.47-minute longer travel time (β = 4.47, 95% confidence interval, CI = 2.59-6.35, p < 0.01), while no significant association was found for total transport time (β = -0.65; 95% CI: -3.17 to 1.88; p = 0.62) (Tables 2, 3). Transport from Kochi City was associated with significantly shorter total transport and travel times in both models.

**Table 2:** Results of multiple linear regression analysis for total ambulance transport time Multiple linear regression analysis was used ^†^The t-statistic values for each variable are reported in the third column ^††^Statistical significance was set at a two-tailed p value of <0.05

Parameters	β (95% confidence interval)	t value^†^	p value^††^
Age			
Adults (age ≥15 years)	1.00 (reference)	-	-
Pediatrics (age <15 years)	-0.65 (-3.17 to 1.88)	-0.50	0.62
Sex			
Female	1.00 (reference)	-	-
Male	0.99 (-1.07 to 3.05)	0.94	0.35
Disposition			
Not mild	1.00 (reference)	-	-
Hospitalization	0.13 (-1.92 to 2.18)	0.12	0.90
Department			
Kochi City	-7.92 (-9.99 to -5.84)	-7.49	<0.01
Others	1.00 (reference)	-	-

**Table 3:** Results of multiple linear regression analysis for time from site departure to hospital arrival Multiple linear regression analysis was used ^†^The t-statistic values for each variable are reported in the third column ^††^Statistical significance was set at a two-tailed p value of <0.05

Parameters	β (95% confidence interval)	t value^†^	p value^††^
Age			
Adults (age ≥15 years)	1.00 (reference)	-	-
Pediatrics (age <15 years)	4.47 (2.59 to 6.35)	4.66	<0.01
Sex			
Female	1.00 (reference)	-	-
Male	0.68 (-0.86 to 2.22)	0.87	0.38
Disposition			
Not mild	1.00 (reference)	-	-
Hospitalization	0.16 (-1.35 to 1.69)	0.21	0.83
Department			
Kochi City	-9.98 (-11.53 to -8.44)	-12.7	<0.01
Others	1.00 (reference)	-	-

## Discussion

In this study, we compared the emergency transportation intervals between children and adults with anaphylaxis. There was no difference in the total emergency transport interval between children and adults, but when comparing each specific time interval, there were significant differences in the interval from the emergency call to on-site arrival, the on-site interval, and the interval from on-site departure to hospital arrival.

From the emergency call to arrival at the site, the time difference between children and adults was one minute; therefore, clinical differences would be scant. The on-site interval was three minute shorter for children than for adults. One possible explanation for the shorter on-site interval among children is the rotational pediatric shift system in Kochi City, which may facilitate quicker hospital selection after hours. However, this interpretation is speculative and was not directly assessed in our data. A previous study reported that most children did not receive medications or undergo procedures by the emergency medical service teams, except for oxygen administration [12]. One possible reason for the shorter on-site interval could be the lack of administration of medications or medical procedures at the scene of the emergency.

The greatest difference was observed in the interval from on-site departure to hospital arrival, where the duration was five minute longer for children than for adults. There are 41 emergency hospitals in the Kochi Prefecture, but only seven can provide emergency care for children. In other words, the number of hospitals that emergency teams can choose from is smaller for children than for adults. Therefore, even when pediatric patients are located in urban areas such as Kochi City, longer travel times may still result due to the limited number of pediatric-capable facilities. This hypothesis is consistent with regional disparities in pediatric emergency infrastructure and requires further investigation. Although most of the children in this study were in Kochi City, which should have provided better access to hospitals, the transportation duration was longer. This suggests that hospital proximity alone may not guarantee faster access if facility capability is limited. The five-minute difference could be fatal in the event of airway obstruction. In fatal reactions to anaphylaxis, the median interval to respiratory and cardiopulmonary arrest has been reported as five minutes for drugs, 15 minutes for bee stings, and 30 minutes for food [7]. Reducing transport intervals is important, as early administration of adrenaline may reduce the risk of conversion [13] and prevent delayed reactions [14].

Despite the small number of pediatric emergency hospitals in Kochi City, there was no difference in the total emergency transport interval between children and adults, even though children had shorter on-site stays. While the rotational pediatric shift system may contribute to more efficient hospital selection, the shorter on-site interval in children is likely also influenced by the limited administration of medications at the scene. Previous studies have shown that prehospital adrenaline use in children with anaphylaxis remains low, which may increase the risk of severe or near-fatal reactions if not promptly addressed [15]. However, this conclusion is tentative and based on indirect inference.

The most common age group in this study was 65-69 years, followed by the 0-4 years age group. Most cases of anaphylaxis have been reported in children aged less than five years [4,16]. In Japan, emergency life-saving technicians have been allowed to administer prescribed AAIs under medical control since 2009. However, AAIs were most often administered to children by their mothers and rarely by emergency life-saving technicians in Japan [15]. Another study on children with anaphylaxis reported that the reason AAIs could not be administered in the prehospital setting was the lack of prescriptions [17]. Therefore, increased AAI prescriptions for children may allow for early adrenaline administration. Alternatively, the use of a physician-staffed rapid response unit, a vehicle dispatched with an emergency physician to the scene, may help enable earlier administration of adrenaline in pediatric cases. Although not evaluated in this study, such systems could be explored in future research. These findings underscore the need to assess and optimize triage protocols and transport strategies, particularly for pediatric patients requiring time-critical interventions such as adrenaline administration.

The strength of this study lies in the fact that the ambulance teams used dedicated tablets to accurately and consistently record key time intervals, ensuring high temporal resolution and minimizing recall bias. This structured and uniform data collection enhances the internal validity and reliability of the findings.

Limitations

This study has some limitations. First, it was conducted within the Kochi Prefecture and was likely influenced by the local emergency transport system. Additionally, as this was a retrospective study using registry data, selection bias may have occurred, and the findings may not be generalizable to regions with different emergency systems or urban infrastructure. However, similar studies have not been conducted in the past; therefore, this study is valuable. Second, the causative agents triggering the allergies were unknown because personal information on each emergency call could not be obtained. Treatment in a prehospital setting, such as AAI use, was not considered. Finally, the present results were based on nonclinical outcome measures, specifically, time intervals, since there were no deaths. The mortality rate for anaphylaxis is low, estimated at 0.05-0.51 per million persons/year for anaphylaxis due to drugs, 0.03-0.32 for food, and 0.09-0.13 for poison [1]. Therefore, it is not possible to compare prognoses in the present study. However, a difference of five minutes indicates that in case of a fatal reaction, there may be a crucial delay in saving lives; therefore, there is room for improvement. Future multicenter or nationwide studies that include prehospital interventions and clinical outcomes would be valuable in validating and extending these findings.

## Conclusions

In this retrospective study, we observed no significant difference in the overall ambulance transport interval between pediatric and adult patients with anaphylaxis. However, the interval from on-site departure to hospital arrival was significantly longer in children by approximately five minutes.

Given the time-sensitive nature of anaphylaxis, in which respiratory or cardiac arrest can occur within minutes, this additional delay could potentially have critical implications for patient outcomes. While our findings suggest an age-related disparity in transport intervals, they should be interpreted cautiously considering the observational design and region-specific context of the study. To mitigate this disparity, future initiatives should consider expanding the availability of pediatric emergency services, refining triage and destination protocols to reduce transport times, and implementing physician-staffed rapid response units, which can deliver early diagnosis and adrenaline treatment at the scene, in pediatric cases. Further multicenter or nationwide studies incorporating prehospital interventions and clinical outcomes are warranted to validate and extend these findings.
